# The Pathophysiology of Perceived Social Isolation: Effects on Health and Mortality

**DOI:** 10.7759/cureus.994

**Published:** 2017-01-24

**Authors:** Adnan Bashir Bhatti, Anwar ul Haq

**Affiliations:** 1 Medical Director of Clinical Research, Spine Surgery, Tristate Brain and Spine Institute, United States; 2 Department of Psychiatry, Capital Hospital, Islamabad, Pakistan

**Keywords:** perceived social isolation, psi, social isolation, loneliness

## Abstract

Perceived social isolation (PSI) is a deficit in normal human social interaction, which has been associated with negative health outcomes. However, the precise mechanisms through which PSI influences human health are not fully known. This review aims at bringing out what is known about these pathways through which social isolation affects human health. We searched PubMed, Medical Literature Analysis and Retrieval System Online (MEDLINE), Excerpta Medica dataBASE (EMBASE), Psychological Information Database (PsycINFO), and Cochrane Library in addition to secondary references from primary journal articles for the most relevant and recent information concerning the research topic. The keywords used were perceived social isolation, loneliness, health outcomes, cardiovascular effects, neuroendocrine effects, depression, and cognitive decline, in animal and human populations. There are clear linkages between PSI and the cardiovascular system, neuroendocrine system, and cognitive functioning. PSI also leads to depression, cognitive decline, and sleep problems. The mechanisms through which PSI causes these effects are neural, hormonal, genetic, emotional, and behavioral. The effects of PSI on health are both direct and indirect. There is a complex interconnected network of pathways through which PSI negatively influences health. These hypothetical pathways using which the effects of PSI have been explained form the base on which further analyses can be carried out.

## Introduction and background

The concept of perceived social isolation (PSI) has been receiving a lot of attention from researchers recently, especially as a result of the advent of increasing social disconnectedness and increasing life expectancy, which has led to rising populations of elderly people [1]. Humans are a social species and so thrive on a wide variety of social interactions and networks. The social characteristics of most species have been described to reflect a broad range of affiliative behaviors which vary in manner and complexity according to the type of species [1-2]. It has been pointed out that sociality hinges on a set of behaviors that enable or impede the initiation and maintenance of social relationships [3]. The occurrence of deficits in these relationships affords researchers the opportunity to measure and evaluate how significant social interactions are to human health and fitness.

An instance of how important social relationships are to human survival is the relationship between a mother and her child and how vital this interaction is for the child’s fitness. This type of relationship is just the beginning of a lifelong process of initiating and maintaining one-on-one and group relationships that develop through the different developmental stages [3].

An understanding of the importance of social interactions to human functioning also leads to the question of what happens when these interactions or relationships are not present or functional. This has driven inquiries to a relatively novel concept, PSI, which has also been referred to in many studies as ‘loneliness’ [3-4] and a perceived lack of social support [5]. It merges the subjective feeling of loneliness and the objective deficiencies associated with social interactions and networks [3].

Various factors account for why an individual becomes socially isolated. Risk factors such as health problems, disability, gender discrimination, loss of loved ones (spouse), living alone, reduced social networks, poverty, and aging have been associated with the occurrence of social isolation [6]. When the individual develops the subjective feeling of being isolated, what happens to the health of the individual? This question has been a central concern for research in health. It also brings to the fore a number of animal studies that have tried to elucidate the health effects of loneliness. These studies have shown how significantly social isolation can affect the health of mammals. Even though taking an animal out of its social group and making it exist alone cannot be compared adequately to the pain of human PSI, it nevertheless suggests how dramatic and significant the effect of absent or inadequate social interaction can be. These animal models have been of great value in helping researchers understand the pathways through which social isolation affects the human body and health.

The relationship between PSI and cognitive decline in the elderly has been pointed out by a few studies that have shown that loneliness is indeed a predictor and marker of pathological brain changes in this group of people [7]. In younger adults, loneliness has been associated with poor sleep habits, increased risk of depression and suicides, and a greater risk of cardiovascular disorders [8-9]. In addition to adults, children and adolescents have also been shown to be susceptible to the negative effects of social isolation. Children are at a greater risk of depressive symptoms, poor sleep, and impaired executive functioning when they face prolonged periods of social isolation [8, 10].

A large part of these studies have focused on the sleep and mental health effects of PSI, despite evidences suggesting that the effects of social isolation cut across every aspect of human psychological and physiological functioning [3]. To further bolster this, a significant volume of research has highlighted the link between social isolation and poor health, including depression, inflammation, and cardiovascular disease [11-13]. The risks associated with social loneliness are therefore obvious. However, the mechanisms through which these risks occur, and the complexities associated with these negative health outcomes, cannot be captured with a mere measure of prevalence or statistical association. It is then necessary to determine whether there are actual pathological linkages between the social concept of loneliness and human physiology rather than just mere statistical findings.

This then becomes the focus of this review: to find out what health outcomes are possible with PSI and to identify the pathways through which PSI affects human health.

### Methods

To access the most relevant content, studies containing the following keywords were sought: PSI, loneliness, health outcomes, cardiovascular effects, neuroendocrine effects, depression, and cognitive decline, in varying animal and human populations. The following abbreviations were used: PSI (perceived social isolation) and CVD (cardiovascular disease).

### Data sources

We used the following sources: MEDLINE, EMBASE, PsycINFO, and Cochrane Library. We also extrapolated from the references from each primary source and searched for additional relevant journal articles. We focused on articles written in or translated into English. A full online search was also conducted using Google Scholar, and where necessary, authors were contacted.

### Eligibility criteria

We incorporated articles that provided precise data on PSI and its association with varying health outcomes. We did not use the size of subject populations, the type of study design, or the specific outcomes of the studies to screen any research. We included studies in which PSI or social isolation was the focus and outcomes were defined as physiological or physical, emotional, cognitive, genetic, cardiovascular, neuroendocrine, and psychological.

The selected journal titles and abstracts were screened by the authors, and those that did not fit the inclusion criteria, or in which there was no consensus between the authors, were discarded.

## Review

One common conclusion in many studies that have examined the deleterious health effects of social isolation is that there is a direct relationship between PSI and morbidity/mortality, or that there is an inverse relationship between social support and morbidity/mortality [14-16]. Some other studies have gone further to point out precisely how risk factors such as obesity, overeating, and diabetes contribute to mortality [3].

### Cardiovascular effects of PSI

One of the most prevalent killers of middle-aged and elderly individuals in the United States is cardiovascular disease [17]. Social isolation has been implicated as a predictor of increased morbidity and mortality due to cardiovascular disease (CVD) [18]. A few epidemiological studies have shown that individuals with poor social support are prone to having hypertension, coronary artery disease (CAD), or cardiac failure [11, 12, 17, 19]. For those who already have one or more of these conditions, a deficiency in social support has also been associated with a faster progression of atherosclerosis, greater risk of major cardiovascular events such as a stroke or myocardial infarction, and doubled risk of CVD mortality [17, 19].

Experimental studies on animals have established that socially isolating animals places them at an increased risk for obesity and type 2 diabetes mellitus [20]. These same animals also face increased risk of developing CVD, inappropriate inflammatory responses, and an unnecessarily augmented response to stress [21]. The exaggerated stress response may put extra load on the heart and systemic vasculature, contributing in a great way to organ damage. Correlating with human participants, research has shown that subjects with poor social support have exaggerated blood pressure and heart rate responses to stressful situations with a remarkably slow recovery time [22]. These same set of people also experience autonomic imbalances [23], exaggerated inflammation [24], increased calcification in the coronary arteries [25], and significant hypertrophy of the left ventricle [26]. These evidences support the hypothesis that social isolation contributes to CVD morbidity and mortality.

### Neuroendocrine effects of PSI

Extant data from human research suggest that there is a relationship between PSI and abnormal activity of the hypothalamic-pituitary-adrenocortical (HPA) axis which is a major component of the endocrine system [27]. An association between loneliness and increased blood levels of catecholamines has also been suggested [12, 28]. As far back as 1984, loneliness studies have shown a poorer competence of the cellular immune system as evidenced by considerably high levels of Epstein-Barr virus (EBV) antibody titers in specific groups of medical students who experience prolonged periods of social isolation [29].

Further confirming the role of the HPA axis in mediating the effects of loneliness on the neuroendocrine system, a study showed that non-psychotic psychiatric inpatients who experienced loneliness had higher urine levels of cortisol than inpatients who had experienced more social support [30]. This result was independent of the influence of stressful life events, as that did not have any impact on the findings. It is important to note that loneliness in this case was self-reported, a parameter that was used to segregate the inpatients into either high or low groups based on self-reported scores.

Another study which measured cortisol levels in the saliva of undergraduate students found that there was a positive correlation between loneliness and salivary cortisol levels, although this correlation became statistically significant only when the loneliness was chronic [31]. In this study, the time the students spent alone did not have any influence on the outcome. Other researchers have confirmed the positive association between PSI and salivary cortisol levels [12, 28, 32].

Besides the influence of loneliness on glucocorticoids levels, research has also gone further to investigate its association with glucocorticoid resistance. First, animal models suggested that negatively perceived social factors and isolation can trigger glucocorticoid resistance, where there is poor response to glucocorticoid signals transduced via glucocorticoid receptors [33]. In humans aged 54 years and above in Taiwan, it has been shown that PSI is associated with smaller neutrophil to monocyte and neutrophil to lymphocyte ratios, which are consistent with glucocorticoid resistance in white blood cells [34].

### Genetic effects of PSI

The genetic effects of social isolation has been revealed through genome microarray analyses which have pointed out the following genetic responses: a reduction in gene expression for genes which modulate glucocorticoid production and response effects; an upregulation of proinflammatory mRNAs (leading to increased production and activation of proinflammatory cytokines and other mediators), and a downregulation of inflammatory regulatory markers in adults (middle- and older-aged) who experienced significant periods of loneliness [35]. 

### Psychological/behavioral effects of PSI

The linkage between loneliness and depression has been established in several studies. In a group of 296 British children who were followed up at ages five, nine, and 13, it was found out that early childhood loneliness (at ages five and nine) was predictive of significant depressive illness later in life [36]. More recent studies measuring ‘cumulative relationship risk’ in middle-aged individuals showed that exposure to factors such as loneliness, low parental support, instability in romantic relationships, and intimate partner violence, correlates with the occurrence of depressive symptoms, with the dose response increasing progressively depending on the number of risks faced by the individual [3, 37].

PSI has also been associated with suicidal ideation in adults, where those who frequently experienced loneliness were at 21% increased risk of having suicidal thoughts (as against 2.5% of those who were not as frequently lonely) and had a 8.4% chance of attempting suicide as against 0.7% for those who were less frequently lonely [38]. These results were recorded in a sample of more than 19,000 individuals older than 14 years.

In elderly individuals, loneliness has been linked with cognitive deficits and later dementia [3, 39-42]. These effects significantly reduce the individual’s quality of life and make maintaining social relationships more difficult – leading to a vicious cycle. In a cohort of 70-year-old individuals, a significant inverse relationship was found between the intensity of loneliness and memory, processing capabilities, and general cognitive ability [40]. Other studies have also predicted rapid cognitive decline and increased risk of developing Alzheimer’s disease in adults [41]. In addition, there is an impairment of executive control in older adults as a result of reduced support. Evidences from self-reports have shown a link between loneliness and reduced effort at expressing positive emotions – a maladaptive response that makes the regulation of other self-controlled behaviors difficult [3,42]. This partly explains the reduced inclination in these older adults to engage in healthful physical activities [43].

### Other effects of PSI

Sleep is deficient in quality in individuals who are deprived of social support [3]. In a research conducted on a group of lonely young adults, it was found that they exhibited significant amounts of restless sleep, indicated by the amount of wakeful periods during sleep [44]. Unlike the effect of loneliness on the quality of sleep, the effect of social isolation on the duration of sleep has been found to be negligible [3]. Nonetheless, lonely individuals still have to contend with daytime dysfunction and fatigue as a result of the less restful sleep they got during the night [45].

### Pathophysiology of the effects of PSI

The effects of PSI on the health of individuals are difficult to discuss singly. This is because the human body functions as a holistic combination of different organs and systems working together to ensure the survival and fitness of the individual. Likewise, the effects of loneliness on health are all-encompassing; it affects multiple organ systems, often together, as the results of the various studies presented earlier have shown [11]. PSI is associated with effects on the cardiovascular, neuroendocrine, and central nervous systems as well as genetic mechanisms and mental health. Comparatively, it is difficult to identify which system suffers the most, as existing information suggests that so many factors complicate physical and mental health outcomes [5]. However, from the standpoint of the affected individual, what is most important is the mechanisms through which PSI leads to worse health and how these can be tackled [11].

In theory, humans have an instinctive need to belong; this need is as basic to human functioning and survival as the need to obtain food, water, and shelter [46]. When this need fails or is not satisfied, there are internal reactions which range from physiological to neurological and psychological [17]. These reactions have been compared with human bodily responses to physical and psychological pain with particular reference to the utilization of the same neurological substrates to mediate these responses [47]. The initial goal of activating this response system is to prevent potential social death, but this objective also leads to deleterious effects on the functioning of the cardiovascular, neurological, and cognitive systems [17].

The effect of PSI on the cardiovascular system has been hypothesized to be both biological and psychological/behavioral. Studies have suggested that individuals who have significant social support and networks have at their disposal tangible resources that can help them live and promote a healthy lifestyle [17]. These individuals tend to be more active and are less likely to engage in risky health behaviors such as excessive consumption of alcohol, smoking, or maintaining poor eating habits [48]. On the other hand, individuals who have less social support tend to take risky decisions and are also at an increased risk of death from cardiovascular emergencies due to the absence or lack of close individuals who can get them the prompt medical attention they need at such times [49]. In addition, the presence of social support is known to serve as a stress-buffer – a way to take one’s mind off stressful life situations and reduce physiological stimulation and overall allostatic load [17].

The biological evidences of the influence of loneliness on cardiovascular health come from animal models which showed a significant increase in the risk of type 2 diabetes mellitus, obesity, abnormal inflammatory responses, and augmented stress reactivity in caged animals [20]. However, in humans, it has been shown that prolonged exposure to stress puts a lot of load on the cardiovascular system as a whole and contributes to the development of target organ damage [17]. More so, lonely human subjects exhibited slow recovery from exaggerated blood pressure and pulse rate responses to perceived stress in addition to other evidences such as significant left ventricular hypertrophy, autonomic imbalance, increased systemic inflammation, and the rapid calcification of coronary arteries [22-26].

**Figure 1:**
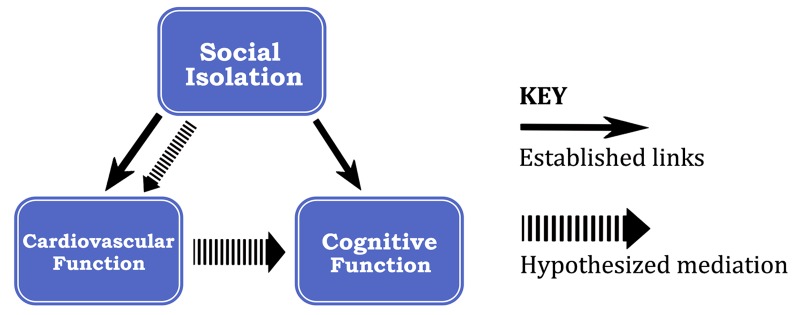
Established and hypothesized pathways between social isolation, cardiovascular function, and cognitive function.

Going further, research has suggested that there is a link between cardiovascular health and cognitive function with respect to the effects of PSI. Particularly, White, et al. presented a model that hypothesizes that PSI directly affects cardiovascular functioning and also affects cognitive functioning by decreasing cerebral blood flow and inhibiting neurovascular coupling [17]. These effects lead to overall deficits in neural integrity and cognitive functioning. Even though this model is yet to be tested, it presents a valid theoretical rationale for the decline in cognitive functioning which has been found in many elderly patients with PSI (Figure 1).

On the neuroendocrine effects of PSI, there is evidence supporting the fact that loneliness is associated with the hyperactivity of the HPA axis and increased blood levels of catecholamines [27, 28]. Both human and animal studies have found higher levels of HPA activity as a result of loneliness, even though the strength of this association has been said to depend on factors such as the time of day when the measurements were made, the duration of loneliness, the type of tissue assayed, the parameters used to evaluate HPA activity, and the reliability of measurements [35].

Loneliness has been positively correlated with salivary and urinary cortisol levels in humans. Overproduction of cortisol negatively affects the physiological functions which the hormone mediates – body metabolism, glucose control, apoptosis, inflammatory regulation, immunity, reproduction, and cardiovascular activity. Even though the body regulates the production of cortisol through negative feedback mechanisms, social factors such as the deprivation of social support have been associated with glucocorticoid resistance, in which the efficiency of glucocorticoid receptors at transducing endogenous signals and mediating negative feedback becomes reduced, leading to pathologic inflammatory processes that contribute to the pathogenesis of diseases such as atherosclerosis, type 2 diabetes, neurodegeneration, and tumor generation [33, 35].

The link between depression and PSI has been well established by a number of studies [36-38]. Theoretically, loneliness and depression have been conceptualized as two overlapping negative and unpleasant states that are distinguished on the basis of the feelings toward social connections [50]. Even though there is limited information regarding the pathways through which loneliness leads to depression, significant findings back up the existence of loneliness as a predictor of depressive symptoms [3].

It is clear that multiple biological and psychological pathways link PSI to health outcomes. PSI leads to acute neuroendocrine changes that influence cardiovascular and other physiologic responses in a way that causes unwanted physical symptoms. How factual these hypotheses are remains the subject of future studies as no evidence has precisely elucidated the causal roles of PSI in humans.

## Conclusions

This study has established the associations between PSI and the etiology and pathogenesis of illnesses – cardiovascular, inflammatory, neuroendocrine, cognitive, and affective disorders. It is clear that the lack of social support influences a wide range of internal processes that result in deranged and inappropriate responses leading to disease. In general, the effects of lack of perceived social support on health can be direct or indirect, which is mostly detrimental. This study has also succeeded in pointing out that there is no single separate way in which social isolation negatively influences human health, but that PSI affects physiologic and psychological functioning in a complex dynamic manner.

The studies examined in this research have provided evidences linking PSI to increased morbidity and mortality. Individuals who face prolonged periods of loneliness are at greater risk of cardiovascular disease and cardiovascular emergencies; they face unnecessarily augmented inflammatory responses, are prone to cognitive decline, and also have to deal with a wide range of metabolic and hormonal imbalances. However, the exact nature in which each of these deficits occur needs to be further elucidated as there are large gaps in knowledge concerning the psychological, behavioral, and physiological mechanisms through which these deficits take place. In addition, considering that a significant proportion of these evidences are based on animal models, it is necessary to find out how humans respond to loneliness states.
